# Assessment of Awareness, Attitudes, and Practices Regarding Environmental Pollution Caused by Single-Use Plastic Bags: A Cross-Sectional Study

**DOI:** 10.7759/cureus.82904

**Published:** 2025-04-24

**Authors:** Jeffrey Joseph, Mamata Subhakar R, Stephen T, Krishna Prasanth, Subhashini Viswanath

**Affiliations:** 1 Department of Community Medicine, Sree Balaji Medical College and Hospital, Bharath Institute of Higher Education and Research, Chennai, IND; 2 Department of Ophthalmology, ACS Medical College, Dr. MGR University, Chennai, IND

**Keywords:** change in behaviour, climatic change, community study, plastic pollution, receptiveness

## Abstract

Introduction

Single-use plastics like bags, bottles, and straws are commonly used, but they degrade slowly, causing major hazards to the environment. Despite efforts such as recycling and bans, their use remains high. Toxic chemicals produced during degradation endanger humans, marine life, and ecosystems, emphasizing the absolute need for effective plastic waste management strategies.

Methodology

This community-based cross-sectional study included 320 participants from the field practice area of a tertiary care hospital in Chengalpattu. Participants were selected using a simple random sampling technique. Data collection was conducted using a pre-tested, semi-structured questionnaire. The collected data were analyzed using Microsoft Excel 2019 (Microsoft® Corp., Redmond, WA) and IBM SPSS Version 21 (IBM Corp., Armonk, NY).

Results

Only 42% of participants were aware of single-use plastics. Lower socioeconomic status and living in semi-pucca or kutcha houses were linked to non-receptiveness toward reducing plastic use. While 47% had started reducing usage, about 52% had adopted sustainable alternatives to combat plastic pollution.

Conclusion

The study identified limited awareness of single-use plastic bags, with usage patterns influenced by socioeconomic status and housing type. A significant number of participants showed non-receptiveness toward reduction efforts, and only a few acknowledged the environmental impact. These findings emphasize the need for enhanced awareness campaigns and targeted interventions to encourage sustainable practices and reduce plastic consumption.

## Introduction

Single-use plastics (SUPs) are the plastics that are intended to be used only once before being thrown away or recycled. It includes things like plastic bottles, food packaging bags, straws, and others [1]. SUP bags have become an integral part of human life in terms of packaging, protecting, and distributing consumer goods [2]. The SUPs, like plastic bags, disposable bottles, plastic wraps, ice cream tubs, gloves, masks, cutlery, are most commonly made from polyethylene (PE) and polypropylene (PP) [3]. Given that plastic materials are difficult to decompose, the increasing amount of plastic in packaging has a direct effect on the environment [4].

Globally, about seven billion tons of plastic are produced, of which only 10% is recycled, and 36% of the plastic is used in packaging for foods and beverages. Approximately 500 billion SUP bags are used every year [5]. The Asia-Pacific region has contributed about half of the world’s plastic production, accounting for about 52%. China contributes about 32% of the global production of plastic materials [6]. India is ranked third worldwide, generating about five million tons of SUP waste, and is positioned 94th in per capita SUP waste at 4 kg annually [7].

Hazardous metals, such as cadmium and lead, can seep into groundwater due to the improper disposal of plastic bags on land. Garbage combined with plastic bags disrupts waste processing equipment and poses issues for landfills. It is a myth that plastic can be disposed of by means of recycling, burning, or land filling because it does not decompose bacterially. Plastic bags take hundreds of years to decompose in landfills [8]. The micro-plastics (0.1 μm to <5 mm) present in the SUP bags pose a bigger threat to humans in the form of chemicals in micro-plastic-contaminated foods. These toxic chemicals present in the plastics, when degraded, are harmful to humans as well as marine life and the environment [9]. India has made several attempts to address the negative impacts of the usage of plastics by recycling and banning the production of one-time use plastics. Even after the ban, which was implemented in 2022 [10], the usage of SUP bags is high in India.

To create a better environment for all, it is important for the community to realize the negative impacts of plastic and limit the use of plastic products in everyday life. Many people, particularly in developing and underdeveloped nations, continue to rely on these SUP bags despite knowing of their adverse effects. The disparity between awareness and behavioral change highlights the necessity for conducting this study. Most of the previous studies have highlighted the awareness, attitudes, and practices of SUP bags, but here in this study, we will be seeing the action and maintenance of the person’s usage practices. The increasing environmental pollution caused by SUP bags has become a significant global concern. This study aims to assess the levels of awareness regarding the environmental impact of SUP bags, examining how well individuals understand the consequences of their usage. Additionally, it explores public attitudes toward SUP bags and the implementation of plastic bans, analyzing the extent of acceptance and resistance to such policies. Furthermore, the study evaluates whether the participants are receptive to the usage of SUP bags, identifying key factors that influence consumer choices. The findings will provide insights into promoting sustainable alternatives and effective policy measures.

## Materials and methods

This community-based cross-sectional study was conducted over a period of three months from December 2024 to February 2025 in the field practice area of a tertiary care hospital located in Anakaputhur, Chengalpattu district. The study area covered Anakaputhur, which has a total population of 42,597 and 11,776 households. The sample size was determined based on a previous community-based cross-sectional study by Joseph et al. [11], which reported that 71.6% of the population was aware of SUP bag usage. Using this prevalence, with an absolute precision of 5%, the estimated sample size for the study was calculated to be 320. A simple random sampling technique was employed for participant selection. Individuals aged 18 years and above residing in urban areas were included in the study. Informed written consent was obtained from the study participants. Ethical clearance was obtained from the Institutional Human Ethical Committee (002/SBMCH/IHEC/2023/2092) of a tertiary care hospital. A list of all households was obtained from the staff of the Urban Health Training Center, and 320 households were randomly selected. The study subjects included the head of the household, their spouse, or any available family member above 18 years of age at the time of the visit, with only one person being interviewed per household. A pretested, semi-structured questionnaire was developed based on a review of the literature (Appendices). A pilot study was conducted among 32 participants, and based on expert opinions in public health, necessary modifications were made to the questionnaire. The participants from the pilot study were excluded from the final analysis. The questionnaire’s reliability was assessed, yielding a Cronbach’s alpha of 0.93, confirming its validity. The validated questionnaire was then used to collect data on awareness and stages of change regarding SUP bag usage. Data were collected, entered, and analyzed using Microsoft Excel 2019 (Microsoft® Corp., Redmond, WA) and IBM SPSS Version 21 (IBM Corp., Armonk, NY). Descriptive statistics were presented using numbers and percentages. The association between demographic variables, awareness, and stages of change related to SUP bag usage was assessed using analytical statistics like chi-square, and the odds ratio was used to establish the statistical significance (at 95% confidence interval). Binary logistic regression analysis was performed to identify independent predictors of SUP bag usage.

## Results

The majority of the study participants (195 (60.9%)) are between the ages of 18 and 24, with 48 (15%) aged over 41. Females constitute 214 (66.9%) of the study population, substantially greater than males (106 (33.1%)). About 85 (26.6%) of participants are married, while the majority (220 (68.8%)) are single. According to the modified Kuppuswamy scale classification [12], 99 (30.9%) of individuals live in the upper middle class, 83 (25.9%) are upper lower class, and 10 (3.1%) are in the lowest class.

Stable living conditions were shown in the finding that 260 (81.3%) of people live in pucca houses. The majority of households are nuclear (162 (50.6%)), followed by three-generational homes (117 (36.6%)). The most significant religion is Hinduism (209 (65.3%)), with Muslims (65 (20.3%)) and Christians (46 (14.4%)) being represented. Two hundred sixty-eight (83.8%) of families have fewer than five members, according to family size as shown in Table 1.

**Table 1:** Sociodemographic details of study participants (N = 320)

S. no.	Variables	Frequency (N = 320)	Percentage (%)
1	Age		
	18-24 years	195	60.9
	25-32 years	47	14.7
	33-40 years	30	9.4
	>41 years	48	15
2	Gender		
	Male	106	33.1
	Female	214	66.9
3	Marital status		
	Single	220	68.8
	Married	85	26.6
	Widowed	15	4.7
4	Modified Kuppuswamy classification (2024)		
	Upper	53	16.6
	Upper middle	99	30.9
	Lower middle	75	23.4
	Upper lower	83	25.9
	Lower	10	3.1
5	Type of house		
	Pucca	260	81.3
	Semi-pucca	44	13.8
	Kutcha	16	5.0
6	Type of family		
	Joint	41	12.8
	Nuclear	162	50.6
	Three-generation	117	36.6
7	Religion		
	Hindu	209	65.3
	Muslim	65	20.3
	Christian	46	14.4
8	Number of members in the family		
	Less than five members	268	83.8
	More than five members	52	16.2

**Figure 1:**
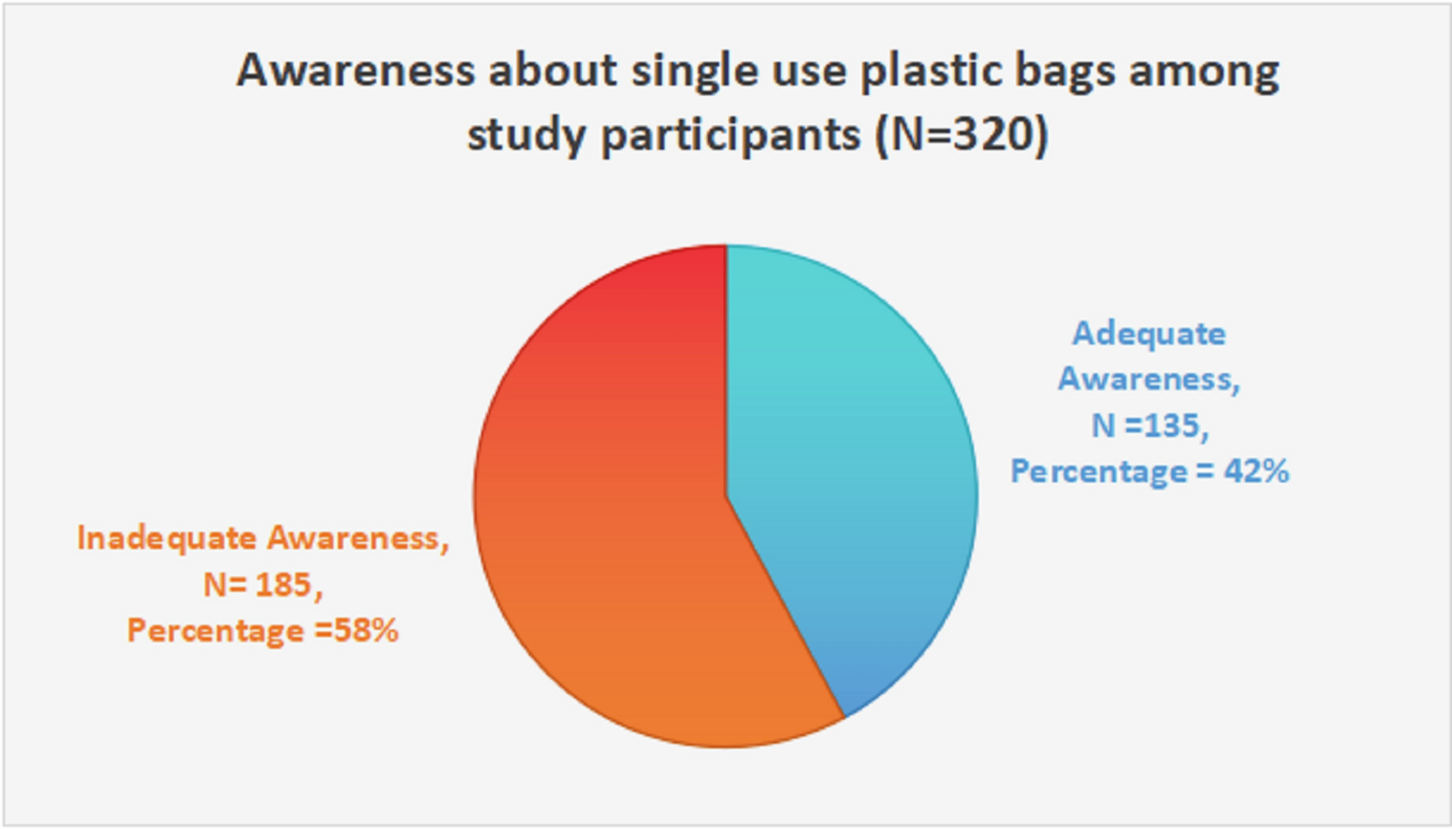
Awareness about single-use plastic bags among study participants (N = 320)

Figure 1 illustrates study participants’ awareness of single-use plastic bags. A significant number, 185 (58%) participants, have inadequate awareness, whereas just 135 (42%) participants have adequate awareness.

Table 2 shows that age has a significant effect on receptiveness, with participants over 33 years showing decreased receptiveness (OR = 1.881, p = 0.016). However, it was not found to be statistically significant after adjusting for the odds ratio (p = 0.937). Stages of change related to SUP bag usage are not considerably affected by gender or marital status. Socioeconomic classification is an important predictor, with lower middle, upper lower, and lower classes being more non-receptive (AOR = 1.891, p = 0.013). Participants who live in semi-pucca/kutcha houses are less likely to be receptive (AOR = 3.636, p < 0.001). Family size, type, and religion have no significant impact on stages of change related to SUP bag usage. These data show that socioeconomic class and housing are important predictors of attitudes against plastic bag use, whereas age appears to be relevant but was not statistically significant after adjusting for the odds ratio.

**Table 2:** Binary logistic regression of attitude and change in behavior toward single-use plastic bags with sociodemographic variables *Represents p-value < 0.05, which is considered statistically significant; unadjusted OR with 95% confidence interval (CI). The significant factors that satisfied p < 0.05 were considered for binary logistic regression analysis, which is represented with adjusted odds ratio (AOR) with 95% CI, and its corresponding p-value at <0.05 is considered statistically significant. Ref - reference category

S. no.	Sociodemographic variables	Attitude and change in behavior toward single-use plastic bags		Total (N = 320), (%)	Unadjusted OR (95% CI)	p-value of unadjusted OR	Adjusted OR (95% CI)	p-value of adjusted OR
		Non-receptive N, (%)	Receptive N, (%)					
1	Age							
	>33 years	39 (12.2)	39 (12.2)	78 (24.4)	1.881 (1.122-3.154)	0.016*	1.025 (0.558-1.883)	0.937
	<32 years	84 (26.3)	158 (49.4)	242 (75.6)	Ref	-	Ref	-
2	Gender							
	Female	80(25)	134 (41.9)	214 (66.9)	0.875 (0.543-1.409)	0.582	-	-
	Male	43 (13.4)	63 (19.7)	106 (33.1)	Ref	-	-	-
3	Marital status							
	Married/widowed	43 (13.4)	57 (17.8)	100 (31.3)	1.320 (0.815-2.137)	0.258	-	-
	Single	80 (25)	140 (43.8)	220 (68.8)	Ref	-	-	-
4	Modified Kuppuswamy classification (2024) [12]							
	Lower middle/upper lower/lower	82 (25.6)	86 (26.9)	168 (52.5)	2.581 (1.615-4.125)	0.000*	1.891 (1.146-3.119)	0.013*
	Upper/upper middle	41 (12.8)	111 (34.7)	152 (47.5)	Ref	-	Ref	-
5	Type of house							
	Semi-pucca/kutcha	41 (33.3)	19 (5.9)	60 (18.8)	4.684 (2.562-8.566)	0.000*	3.636 (1.837-7.198)	0.000*
	Pucca	82 (25.6)	178 (55.6)	260 (81.3)	Ref	-	Ref	-
6	Number of members in the family							
	>5 members	25 (7.8)	27 (8.4)	52 (16.3)	1.606 (0.883-2.921)	0.118	-	-
	<5 members	98 (30.6)	170 (53.1)	268 (83.8)	Ref	-	-	-
7	Type of family							
	Nuclear	61 (19.1)	101 (31.6)	162 (50.6)	0.935 (0.596-1.467)	0.771	-	-
	Joint/three-generation	62 (19.4)	96 (30)	158 (49.4)	Ref	-	-	-
8	Religion							
	Muslim/Christian	42 (13.1)	69 (21.6)	111 (34.7)	0.962 (0.599-1.545)	0.872	-	-
	Hindu	81 (25.3)	128 (40)	209 (65.3	Ref	-	-	-

## Discussion

SUP bags cause immense environmental pollution, although public awareness and change in behavior are minimal. This study examined participants' awareness, attitudes, and practices regarding plastic bag use. The findings show that the majority lack awareness, with socioeconomic class and housing conditions influencing receptiveness. Addressing this issue involves targeted educational initiatives as well as policy-driven measures. This discussion focuses on the ramifications of these findings, highlighting the critical need for sustainable alternatives along with increased public awareness to effectively reduce plastic pollution.

In our study, females (134 (41.9%)) and the younger age group (158 (49.4%)) were more receptive to change toward SUP bags. Those who have better education, income, and housing, according to the modified Kuppuswamy scale classification [12], have better receptiveness toward the change in using SUP bags. A study done by Gannavarapu et al [13] showed that 88% of Gen-Z participants who are relatively younger in age have good awareness, and about 80% are ready to live a lifestyle without SUP bags. Similarly, a study done in Ghana [14] shows that participants with higher incomes and belonging to the upper economic class show better awareness and attitudes toward sustainability. A survey conducted in Nepal [15] has shown that 98% of the participants, who are females (56.1%), are willing to quit plastic usage and use sustainable alternatives. Conversely, in a study done by Northen et al. [16], older respondents over 50 years and above showed more efforts in reducing plastic usage and pollution, which is unlike our study, where younger age people were having more positive practices. The study done by Abd Rahim et al. [17] showed age, education level, and income to be notable disparities in attitudes and practices related to SUPs. Cultural, economic, and generational differences influence people's attitudes about eliminating SUPs. Younger people, affected by the online world activism and trends, exhibit greater adaptability, whereas older generations might adopt sustainability through experience. Education, income, availability to alternatives, policies, and cultural standards all impact attitudes in various nations.

Several studies have examined the awareness and attitudes toward SUP bags, yielding both concordant and discordant findings relative to the reported 58% of participants with inadequate awareness in our present study. In a study conducted in Karnataka [18], more than 70% of participants admitted the negative impact of SUPs on health. However, more than 95% were unaware of their significance in global warming and climate change, suggesting inadequate knowledge. Despite this, 80% supported a ban on SUPs, with more than 60% willing to consider alternatives. However, 82.4% continued to use plastic bags on a regular basis, suggesting an imbalance between perceptions and behaviors. Similarly, a study in Kanchipuram, Tamil Nadu, observed that 71% of participants comprehended the health fears to animals from plastic pollution [19]. In contrast, a study conducted in Perambalur district, South India [20], showed that 92.5% of people used plastic on a regular basis, with 92.6% being aware of plastic alternatives. This shows that despite being aware of alternatives, people prefer to use them. Younger age, male gender, greater education, urban setting, and non-agricultural occupations were all significantly associated with increased plastic use. Disparities between these studies may be due to regional disparities, educational backgrounds, urban versus rural settings, and cultural attitudes toward environmental issues. While awareness levels differ, one constant finding is the inconsistency of information, attitudes, and actual plastic use habits. This emphasizes the need for tailored alternatives that promote awareness and address behavioral change, taking into account the various socio-demographic characteristics impacting plastic usage in diverse groups.

In our study, 75% of the participants had positive attitudes toward the reduction of SUP bags. In a study conducted in South Africa [21], 88% of the participants accepted that SUPs are being overused. However, only 22% have shown that they have avoided those products, indicating a lack of practice. Similarly, another study done in the Philippines [22] has shown that 90% of the participants have positive attitudes toward the reduction of SUP bags. The participants fully supported the newer initiatives and the local bans in the country. A study done in Canada showed that 93.7% of people were personally motivated to reduce the consumption of SUPs. However, the people were not willing to pay more for a sustainable alternative [23]. The similarities in the present study, when compared to other studies, would have arisen due to strong regulations, social norms, and economic factors influencing readiness to adopt alternatives, as seen in Canada, the Philippines, and other studies highlighting various kinds of diligence. While many people support reducing SUPs, actual behavior is determined by convenience, cost, and policy enforcement.

In the present study, about 47% of the participants have started to actively take steps to reduce SUP bag usage, and about 52% have started to use sustainable alternatives to reduce plastic pollution. Similarly, in a study done in the United Arab Emirates by Alteneiji et al., participants showed positive practices in accordance with positive awareness and attitudes toward SUP bags. The participants also willingly use sustainable alternative options over plastic bags [24]. In another similar study in Puducherry, the usage of SUP bags has been drastically reduced from 80.4% to 16.4%. This change was due to a plastic ban, which was implemented, and the people have been receptive to the change and have reduced their use of SUP bags [25]. The similarities could also be attributed to improved environmental education, community-driven initiatives, and individual, family, and societal responsibility activities that promote sustainable alternatives. Media impact, social standards, and economic incentives all help to drive behavior change. Furthermore, government backing, the availability of environmentally friendly approaches, and cultural adaptability all help to reduce SUP bag consumption across areas. Conversely, a study done in Ambon, Indonesia [26] has shown that despite having adequate knowledge about plastic pollution, the study participants have shown that there is a high usage of SUP bags, indicating the need for improved education on sustainability. Also, in contrast, a study done by Devi et al. [27] has shown that younger age and females exhibit pro-environmental attitudes and practices toward SUP bags. However, high education levels do not ensure good practices, as the reduction of plastic bags remains low. This indicates the need for better interventions and alternatives. The concordant results could have been influenced by various determinants like financial constraints, convenience, the accessibility of sustainable alternatives, social influences, government initiatives, and habitual behaviors, which can all play a role in discrepancies. In some areas, dependence on plastic persists due to affordability and a lack of enforcement, highlighting the need for targeted policies and infrastructure improvements.

Limitation

As a cross-sectional study, it is limited in capturing data at a single point in time, and it is difficult to establish the causal relationship between awareness, attitude, and change in behavior. Relying on self-reported data introduces the risk of social desirability and recall bias. The findings may not be generalizable as the sample size is limited and defined to a particular geographic region.

Recommendation

Policymakers should launch focused educational initiatives to highlight the environmental risks of plastic pollution, particularly its impact on air and water pollution and climate change. Tailored interventions must take sociodemographic aspects into consideration, with a particular emphasis on low-income and highly populated housing areas. Implementing subsidies for reusable alternatives, adopting stricter plastic distribution laws, and including sustainability education into school curricula can all help to promote long-term behavioral change. The general population must be empowered through accessible knowledge and community-based activities to embrace ecologically responsible practices and avoid SUP usage.

## Conclusions

The study highlights a significant gap in awareness about the environmental impact of SUP bags. Most participants had inadequate awareness about the adverse effects of plastic bag usage, with socio-demographic factors, such as type of housing and socioeconomic factors, playing a vital role in influencing the usage of SUP bags. Despite the fact that half of the participants were aware, they were still not so receptive to bringing about a change in reducing the usage of SUP bags. Very few were aware that plastic pollution contributes to air and water pollution, climate change, which leads to global warming.

## Appendices

**Table 3:** Questionnaire on single-use plastic *Reference: [12]

Demographic variable	Response options
1. Age	__________
2. Gender	Male/female
3. Marital status	Single/married/widowed
4. Highest education level of head of family*	1. Illiterate 2. Literate, no formal education 3. 1 to 5 standard 4. 6 to 8 standard 5. 9 to 12 standard/diploma post-class X 6. Graduate/diploma post class XII and above 7. Postgraduate/professional degree
5. Occupation of head of family*	1. Senior officials/managers 2. Professionals 3. Technicians/associate professionals 4. Clerks 5. Skilled workers/shop and sales worker 6. Skilled agriculture/fishery 7. Craftsman or related trade workers 8. Machine operators/assemblers 9. Elementary occupation 10. Unemployed
6. Monthly income (Rs)*	1. ≥2,13,814 2. 1,06,850-2,13,813 3. 80,110-1,06,849 4. 53,361-80,109 5. 31,987-53,360 6. 10,703-31,977 7. ≤10,702
7. Type of house	Pucca/semi-pucca/kutcha
8. Number of members in the family	__________
9. Type of family	Joint/nuclear/three-generation
10. Religion	Hindu/Muslim/Christian
Question	Response (yes/no)
1. Are you aware that single-use plastic bags contribute significantly to environmental pollution?	Yes / No
2. Are you aware that plastic bags contribute to water and air pollution, harming human health?	Yes / No
3. Do you know that burning plastic waste releases harmful gases that affect the respiratory system?	Yes / No
4. Are you aware of the risks of single-use plastic bags contaminating food & water, affecting climate change, and disturbing the marine ecosystem?	Yes / No
5. Have you heard about any government policies, regulations, or bans on single-use plastic bags in your area?	Yes / No
6. Do you believe single-use plastic production contributes to climate change and global warming?	Yes / No
Question	Response (yes/no)
1. Do you think you have no problems regarding SUPs that need changing?	Yes / No
2. Does it make sense to you to reduce SUP consumption?	Yes / No
3. Do you feel that discussions on reducing SUPs do not apply to you?	Yes / No
4. Do you believe there is nothing you need to change regarding SUP reduction?	Yes / No
5. Do you think you may be part of plastic pollution, but do not believe so?	Yes / No
6. Do you find discussions about plastic pollution boring or unnecessary?	Yes / No
7. Do you feel that you do not need to spend time thinking about SUP reduction?	Yes / No
Question	Response (yes/no)
8. Do you think you might be ready for SUP reduction?	Yes / No
9. Do you believe it might be worthwhile to work on reducing your SUP use?	Yes / No
10. Have you been thinking about using reusable alternatives?	Yes / No
11. Are you hoping that environmental education will help you understand SUP reduction?	Yes / No
12. Do you believe you have a problem regarding SUPs and should work on it?	Yes / No
13. Do you wish you had more ideas on how to reduce SUP consumption?	Yes / No
14. Do you hope someone will provide good advice on reducing SUPs?	Yes / No
Question	Response (yes/no)
15. Are you actively doing something about reducing SUP consumption?	Yes / No
16. Have you started taking steps to avoid excessive packaging with SUPs?	Yes / No
17. Do you find SUP reduction difficult at times, but are still working on it?	Yes / No
18. Are you making efforts to reduce SUPs in food packaging?	Yes / No
19. Are you working on using reusable alternatives to SUPs?	Yes / No
20. Have you started reducing SUPs but need help?	Yes / No
21. Do you believe actions matter more than just talking about SUP reduction?	Yes / No
22. Are you actively working on reducing SUPs?	Yes / No
Question	Response (yes/no)
23. Do you worry that you may go back to using SUPs after stopping?	Yes / No
24. Have you successfully used reusable alternatives but are unsure of maintaining them?	Yes / No
25. Do you feel you are not following through with changes regarding SUPs?	Yes / No
26. Did you expect to be free of SUPs after switching to alternatives, but still struggle?	Yes / No
27. Do you feel you need extra motivation to maintain the changes you’ve made?	Yes / No
28. Are you here to prevent yourself from relapsing into using SUPs?	Yes / No
Question	Response (yes/no)
29. Do you intend to reduce SUP usage soon?	Yes / No
30. Are you willing to pay extra to use reusable alternatives while shopping?	Yes / No
31. Do you intend to avoid SUPs as much as possible while shopping or buying?	Yes / No
32. Are you willing to give up SUPs completely in favor of reusable alternatives?	Yes / No
